# Predictive radiogenomics modeling of EGFR mutation status in lung cancer

**DOI:** 10.1038/srep41674

**Published:** 2017-01-31

**Authors:** Olivier Gevaert, Sebastian Echegaray, Amanda Khuong, Chuong D. Hoang, Joseph B. Shrager, Kirstin C. Jensen, Gerald J. Berry, H. Henry Guo, Charles Lau, Sylvia K. Plevritis, Daniel L. Rubin, Sandy Napel, Ann N. Leung

**Affiliations:** 1 Stanford Center for Biomedical Informatics Research, Department of Medicine & Department of Biomedical Data Science, Stanford University, Stanford, CA, USA; 2 Department of Radiology, Stanford University, Stanford, CA, USA; 3 Thoracic and GI Oncology Branch, CCR, National Institutes of Health, National Cancer Institute, Bethesda, MD, USA; 4 Department of Pathology, Stanford University Medical Center, Stanford, CA, USA; 5 Pathology and Laboratory Service of Veterans Affairs Palo Alto Health Care System, Palo Alto, CA, USA; 6 Department of Radiology, Stanford University, Veterans Affairs Palo Alto Health Care System, Palo Alto, CA, USA

## Abstract

Molecular analysis of the mutation status for *EGFR* and *KRAS* are now routine in the management of non-small cell lung cancer. Radiogenomics, the linking of medical images with the genomic properties of human tumors, provides exciting opportunities for non-invasive diagnostics and prognostics. We investigated whether EGFR and KRAS mutation status can be predicted using imaging data. To accomplish this, we studied 186 cases of NSCLC with preoperative thin-slice CT scans. A thoracic radiologist annotated 89 semantic image features of each patient’s tumor. Next, we built a decision tree to predict the presence of EGFR and KRAS mutations. We found a statistically significant model for predicting EGFR but not for KRAS mutations. The test set area under the ROC curve for predicting EGFR mutation status was 0.89. The final decision tree used four variables: emphysema, airway abnormality, the percentage of ground glass component and the type of tumor margin. The presence of either of the first two features predicts a wild type status for EGFR while the presence of any ground glass component indicates EGFR mutations. These results show the potential of quantitative imaging to predict molecular properties in a non-invasive manner, as CT imaging is more readily available than biopsies.

Non-small cell lung cancer (NSCLC) accounts for 85% of all lung cancer with adenocarcinoma and squamous cell carcinoma comprising the two most common histopathologic subtypes^1^. Besides clinicopathological characteristics such as staging, molecular properties of NSCLC tumors are used to determine treatment of NSCLC. In the current era of precision medicine, mutational testing for NSCLC of selected genes is now standard practice to determine whether affected patients are likely to respond to targeted therapy^2^. This includes testing for mutations of epidermal growth factor receptor (EGFR)^3^, a cell surface receptor activating cell growth and survival, and Kirsten rat sarcoma viral oncogene homolog (KRAS), downstream of EGFR, which activates the same pathway when mutated^4^. A third group is defined by re-arrangements of anaplastic lymphoma kinase (ALK)^5^. These three mutations are generally mutually exclusive^6^. EGFR mutated tumors are sensitive to the tyrosine kinase inhibitors (TKIs) gefitinib and erlotinib, whereas KRAS mutated tumors are not. ALK rearranged tumors are not sensitive to EGFR TKIs, but are sensitive to ALK specific TKIs such as crizotinib^5^.

A recent study has shown that computed tomography (CT) image features are correlated with EGFR mutation status^7^. More specifically, it showed that the proportion of ground glass opacity (GGO), a CT image feature defined as a hazy opacity that does not obscure the underlying structures, is correlated with EGFR mutation status. Similarly, another study demonstrated that tumor location and other image features are correlated with ALK rearrangements^8^. Other advanced imaging studies^9^ have taken a quantitative approach to link imaging with molecular properties of NSCLC^10^,^11^,^12^,^13^,^14^,^15^ and other tumors^16^,^17^,^18^,^19^,^20^,^21^,^22^,^23^. Based on these studies, we hypothesized that a multivariate predictive model of mutation status based on image features would be successful. We specifically investigated if a collection of radiologist-observed qualitative image features can be used to predict the mutational status of NSCLC.

Our results show that a multivariate image signature exists that reliably predicts EGFR mutation presence but not KRAS mutations. Moreover, this image signature outperforms models based on only clinical data and models combining clinical and semantic image features. This opens up interesting opportunities for non-invasive diagnosis and treatment management^24^, and also has the potential to allow *in vivo* monitoring during a course of therapy.

## Results

### Semantic image features show strong correlation with EGFR but not with KRAS mutation status

**Figure 1:**
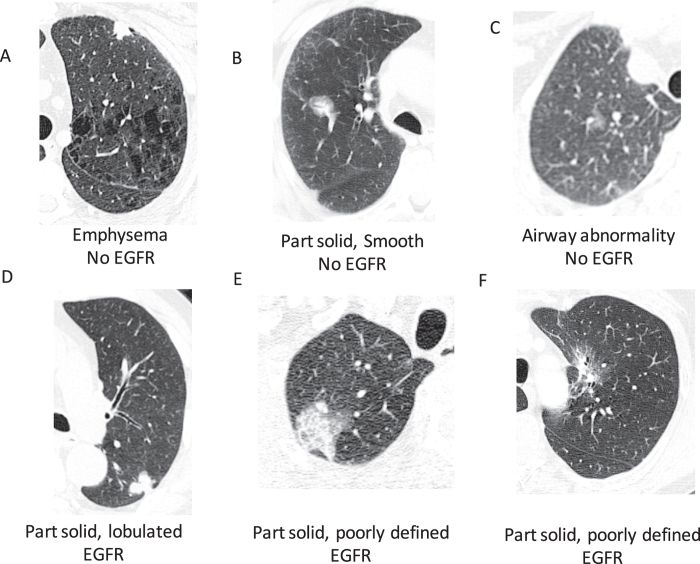
Demonstration of some of the semantic features applied to tumors in our cohort. Note some features (e.g. airway abnormalities, emphysema) are not always depicted on the cross-sections showing the tumor. (**A**) Solid, lobulated squamous cell carcinoma with emphysema, (**B**) part solid, smooth adenocarcinoma, (**C**) ground glass poorly defined adenocarcinoma with airway abnormality, (**D**) part solid, lobulated adenocarcinoma, (**E**) part solid, poorly defined adenocarcinoma, (**F**) part solid, poorly defined adenocarcinoma.

Table 1 shows the clinical characteristics of our cohort of 186 non-small cell lung cancer patients. We found 22% of patients were positive for a mutation in EGFR (either exon 19 deletion or the L858R mutation) and 17% for a mutation in KRAS. Only one patient tested positive for an ALK re-arrangement, excluding ALK from further analysis. We found 16 semantic features were significantly correlated with the presence of EGFR mutation (Q-value < 0.05) whereas no features passed the significance threshold for KRAS (Table 2). Figure 1 shows representative images displaying the important semantic features we discovered in this study. The top predictive features for EGFR are related to the presence of emphysema and the amount of ground glass in the lesion. The presence of emphysema is strongly negatively correlated with the presence of EGFR (Q-value 1.02E-05), whereas the larger the ground glass component of a lesion, the more likely the lesion tested positive for an EGFR mutation (Q-value 6.99E-06). Another observation was that the presence of airway abnormalities is indicative of EGFR wild type tumors. Next, smooth or irregular margins indicate EGFR wild type tumors, whereas larger irregularities such as spiculated, lobulated or poorly defined margins indicate EGFR mutated tumors.

### Multivariate analysis using decision tree modeling predicts EGFR mutation status

**Figure 2:**
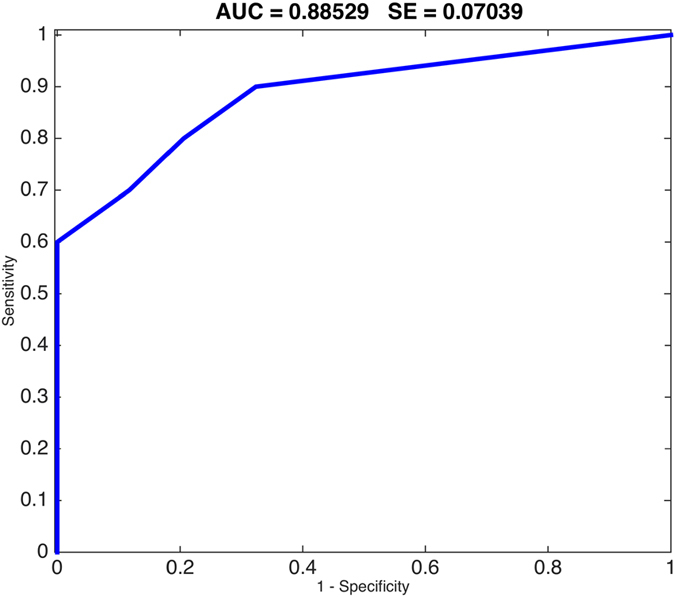
ROC curve showing sensitivity/specificity tradeoff for predicting EGFR mutation status using 5 semantic features.

We used a multivariate decision tree model to predict the presence of EGFR and KRAS mutations. To estimate the performance for predicting EGFR and KRAS, we split the data set (100 times, each with 70% of the samples for training and 30% for testing) in a stratified manner based on smoking, gender and medical center. This resulted in a test set performance of 0.89 AUC for EGFR mutation status prediction (std 0.07, Fig. 2). We next repeated the same analysis using only the adenocarcinoma. This resulted in a similar test set performance of 0.87 AUC for EGFR mutation status prediction (std 0.10). Similar models for KRAS did not result in a useful model (AUC 0.55).

### Clinical data combined with semantic image features does not improve multivariate modeling of EGFR mutation status

We compared our modeling approach with models only using clinical data and with a combination of clinical and semantic image features including all patients with both histologies. Using clinical data only resulted in an AUC of 0.74 (std 0.05), significantly worse compared to the semantic image feature model (P-value < 0.001). The top selected clinical variables were age and smoking status. Combining clinical data with semantic image features did not improve the performance of the semantic feature-only model (AUC 0.82). Note that in only half of the 100 data splits were clinical variables selected for in the model, explaining the similar performance compared to the image feature only model.

### Image feature importance emphasizes the importance of the lesion’s appearance and its environment

The top two features when considering all NSCLC tumors are the presence of emphysema and any airway abnormality. Both features were indicative of non-EGFR mutated tumors (Table 3). Next, increasing irregularity of shape of the nodule margins was indicative of EGFR-mutated tumors. Finally, two features capturing the attenuation of the lesion were predictive of EGFR mutation status. When building a decision tree model only on the subset of adenocarcinoma in our cohort, the top feature ranking was not affected (Table 3).

### A decision tree for predicting EGFR mutation status

**Figure 3:**
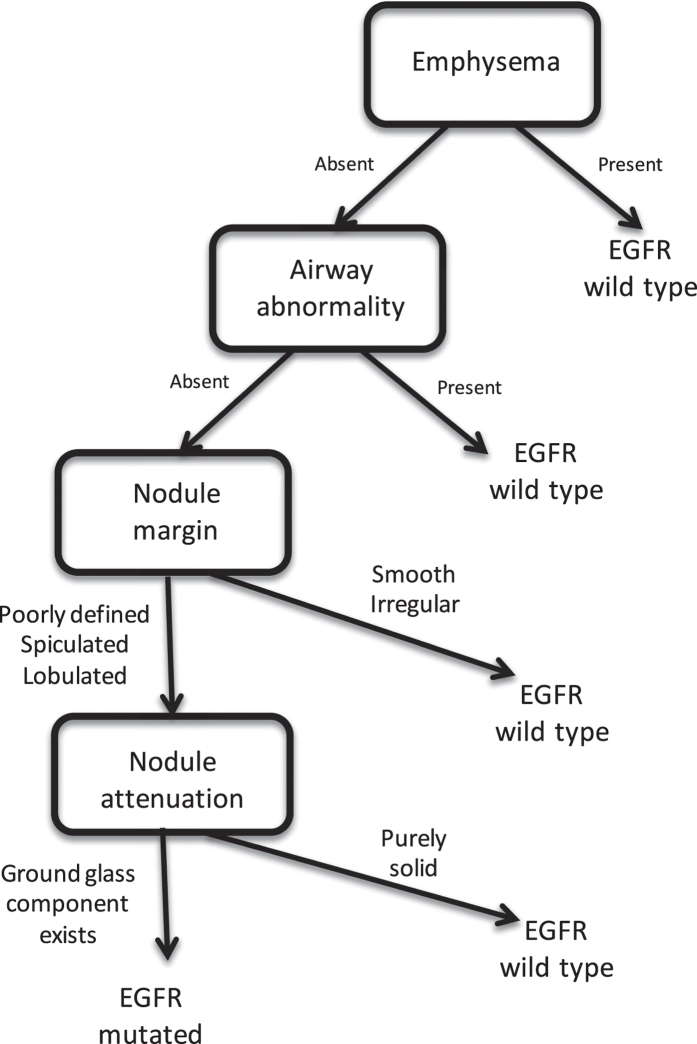
Decision tree for predicting EGFR mutation status using a combination of five semantic image features.

Figure 3 shows the decision tree predicting EGFR mutation status including all patients with both histologies. This model uses only four semantic features. The presence of emphysema is at the root of the tree, determining EGFR wild type tumors, followed by tumors with airway abnormalities also determining EGFR wild type tumors. Next, tumors that have smooth or irregular margins are again predicted to have no EGFR mutation. Next, for the remaining tumors that have lobulated, spiculated or poorly defined margins, if they contain any ground glass component, they are predicted to be EGFR mutated. Finally, for purely solid lesions, when the margins are lobulated, spiculated or poorly defined, the model predicts the presence of an EGFR mutation.

### Inter-reader variability of the decision tree

Finally, we have studied the variability of our decision tree model to different readers. Inter-reader variability ranged from a Cohen’s kappa statistic of 0.13 for nodule attenuation to 0.85 for emphysema (Table 4). Next, we used the additional readers’ annotations in the model computed by using the first reader’s annotations to predict EGFR mutation status, resulting in an AUC of 0.82 and 0.85 for Reader 2 and Reader 3, respectively, which is similar to the performance of Reader 1. There was no statistical difference between the performances of all three readers.

## Discussion

Radiogenomics has the potential to predict molecular characteristics of human tumors by non-invasive methodology. In this study, we have shown the potential to predict EGFR mutation status using a decision tree of semantic image features. This tree uses a combination of four image features to predict the EGFR mutation status. The top features of wild type tumors are related to the presence of emphysema and the presence of airway abnormalities. Next, our analysis also confirms the association between ground glass opacity and the presence of EGFR mutations^7^. In addition, in both univariate and multivariate analyses we observe that certain characteristics of the nodule margins are also indicative of EGFR mutations.

We decided to use a decision tree due to its high degree of interpretability facilitating the possible use of this model in daily practice (Fig. 1). Moreover, decision trees allow extracting specific types of nonlinearities from the data. This was important as regularized logistic regression modeling failed to find a significant performance in our cohort (data not shown). We did not consider black box models, as we choose to have high interpretability of the developed models.

We opted to have the model be useful in the largest possible cohort of NSCLC. Therefore, we focused on most non-small cell lung cancers including also squamous cell carcinoma, which are unlikely to be EGFR mutated. Moreover, although the histopathologic classification is readily distinguishable in tissue samples, it is not always apparent from the imaging phenotype. Next, we excluded certain forms of NSCLC such as central obstructive lesions and pneumonic form lesions. Central obstructive lesions cause obstructive phenomena that are not distinguishable morphologically from the tumor itself. Similarly, pneumonic form lesions present as areas of consolidation that involve some or most of a lung segment or lobe^25^. These lesions do not present as a nodule and thus are not suitable for characterization by our semantic features that were originally designed specifically for the evaluation of nodules.

We were not able to build predictive models for mutations in KRAS. There are several possible explanations for this. First, KRAS mutations were slightly less prevalent in our cohort than EGFR with 17% vs. 22% (Table 1). Another potential hypothesis is that KRAS mutations do not result in radiographic manifestations that can be elucidated with by semantic features to the same extent as EGFR mutations, which seem to have particular observable growth patterns.

Based on these results, our study warrants further investigations. Future work should focus on large-scale multi-center validation studies with the following evaluations. First, we observed variability of the final features between three readers however; we observed no statistically significant difference in the performance estimated by each of the individual readers. Studying the variability amongst radiologists in multi-institutional cohorts is required to further study the robustness of the annotation of semantic features. Second, we chose to use only semantic features for this analysis, whereas other studies have shown the utility of quantitative features computed directly from the image data^10^,^11^,^26^,^27^. Features computed directly from the gray values might reveal patterns that are not obvious to human observers and should be investigated. However, we note that computational analysis of the tumors on the CT scans requires segmentation of the tumors in 3D from the image data, which is still a largely unsolved problem, presenting its own inter-operator/inter-algorithm variability; on the contrary, the selection of a small set of semantic features is something radiologists can do easily during the course of their normal duties. In addition, we have not studied distinguishing the types of EGFR mutation going beyond the diagnostic setting as this could have impact on treatment selection. More specifically, distinguishing exon 19 deletions and L858R point mutations from T790M point mutations and exon 20 insertions after anti-EGFR treatment, could improve treatment management as the former mutations have increased response to tyrosine kinase inhibitors compared to the latter^28^,^29^,^30^.

In summary, we report a multivariate predictive radiogenomics framework that is able to predict molecular characteristics of lung cancers (AUC 0.89) from CT scans in a non-invasive manner. This work motivates large-scale multi-center retrospective and prospective analyses of CT images of lung cancers to provide radiologists and oncologists with additional information at diagnosis and during treatment of NSCLC^24^. It remains to be investigated if this multivariate image signature remains predictive during therapy, as CT imaging is more readily available and less invasive than repeated biopsies during treatment.

## Materials and Methods

### Image data collection and annotation

We collected 196 untreated cases of NSCLC which had preoperative CT scans performed between 4/7/2008 and 09/15/2014 at two medical centers. We excluded 10 cases with pneumonic form or central obstructive lesions, resulting in a data set of 186 tumors. The corresponding CT images were de-identified and an experienced thoracic radiologist (A.N.L.) used ePAD^31^, a publicly-available annotation tool, and annotated each case with a data collection template that specifies 85 semantic image features taken from a controlled vocabulary^32^ (Supplementary Table 1). All variables have binary values reflecting the presence or absence of radiographic features except for four variables that are ordinal in nature. The ordinal features are ground glass opacity (6 classes from 0–100%), size of the solid component (5 classes from pure solid to pure ground glass), emphysema severity (5 classes from 0–100%) and irregularity of the margins (five classes from smooth to poorly defined).

### Clinical data collection

We collected the following clinical variables from each patient: age, histology, sex and smoking status. Histopathologic subtypes consisted of the following subtypes: adenocarcinoma, adenocarcinoma (lepidic predominant pattern), squamous cell carcinoma, and NSCLC, not otherwise specified. Smoking was categorized as never smoker, former smoker or current smoker.

### EGFR and KRAS mutation testing

Mutation testing was done for both EGFR and KRAS using multiplex PCR followed by single nucleotide mutation detection using SNaPshot technology based on dideoxy single-base extension of oligonucleotide primers^33^. For EGFR, exons 18, 19, 20 and 21 were tested, and for KRAS missense mutations with amino acid substitution at positions 12 or 13. Mutations were combined irrespective of their location in the tested exons.

### Univariate analysis

We used univariate analysis to investigate the association of image features with the presence of EGFR and KRAS mutations. We used the Wilcoxon rank sum test in combination with the False Discovery Rate (FDR) to correct for multiple testing^34^. We reported the Q-value defined as the proportion of false positives incurred when the Wilcoxon test is significant at the 0.05 level.

### Predictive modeling using decision trees

We built a predictive model using image features to predict the presence of EGFR and KRAS mutations using a classification tree^35^. We used pruning, a technique to reduce a tree by turning a branch node into a leaf node and moving this leaf node under the original branch. We used an optimal pruning scheme that first prunes branches giving the least improvement in training accuracy^35^. Each leaf of the tree had to have at least five observations in that leaf node.

### Comparison with clinical data

We compared the models based on image features with decision trees using only clinical data and decision trees using a combination of image features and clinical data. We used the Wilcoxon rank sum test to compare the performance of image feature models with models using only clinical data and models using the combined clinical and image data.

### Model building strategy and validation

We split our data set 100 times 70% for training and 30% for testing in a stratified manner to estimate generalization performance. We stratified this split based on smoking, gender, histology and medical center. We estimated the performance of the model using the area under the receiver operating characteristic curve (AUC).

### Inter-reader variability of semantic features and EGFR prediction

To study the inter-reader variability of the selected model, two additional readers (H.H.G. and C.L.) provided annotations for the selected features used by the selected model. We used Cohen’s kappa statistic to estimate the variability of annotations by different readers. Next, we estimated the performance of the model for each of the additional readers’ annotations. We statistically compared the AUC estimates for each of the three readers using a statistical test to compare ROC curves^36^.

### Ethical approval

The study was approved by the Institutional Review Board (IRB) of Stanford University. Informed consent was obtained from all individual participants included in the study and all the experiments described here were performed in accordance with the approved guidelines.

## Additional Information

**How to cite this article**: Gevaert, O. *et al*. Predictive radiogenomics modeling of EGFR mutation status in lung cancer. *Sci. Rep.* **7**, 41674; doi: 10.1038/srep41674 (2017).

**Publisher's note:** Springer Nature remains neutral with regard to jurisdictional claims in published maps and institutional affiliations.

## Supplementary Material

Supplementary Table 1

## Figures and Tables

**Table 1:** Clinical data for the NSCLC cohort (N = 186).

	Number	Percentage
**Sex**		
Male	120	65%
Female	66	35%
**Histology**		
AdenoCarcinoma	153	82%
AdenoCarcinoma (BAC)	1	1%
Squamous cell carcinoma	29	16%
NSCLC	3	2%
**Smoking**		
non smoker	42	23%
former smoker	113	61%
current smoker	31	17%
**Location**		
Academic center	113	61%
VA	73	39%
**EGFR**		
positive	40	22%
negative	110	59%
missing	36	19%
**KRAS**		
positive	32	17%
negative	118	63%
missing	36	19%

**Table 2:** Univariate correlation of EGFR mutation status with semantic image features.

Semantic feature	Test	P-value	Q-value
Emphysema: Presence	Fisher exact test	6.26E-09	4.02E-07
Primary Emphysema Laterality: Both	Fisher exact test	1.09E-08	3.50E-07
Overall Emphysema Severity: Multi-class with increasing % of emphysema	Spearman rho	1.98E-08	4.23E-07
Ground glass category: Multi-class with increasing % of GGO	Spearman rho	2.20E-08	3.53E-07
Primary Distribution: Upper predominant	Fisher exact test	8.84E-08	1.14E-06
Lung Parenchyma Features: Presence of airway abnormality	Fisher exact test	3.76E-07	4.02E-06
Nodule Internal Features: Presence of reticulation	Fisher exact test	1.96E-05	0.00017956
Overall Emphysema Severity: Low severity (1–25%) vs. rest	Fisher exact test	2.75E-05	0.00022074
Nodule Attenuation: Solid	Fisher exact test	4.99E-05	0.00035601
Nodule Periphery: Normal	Fisher exact test	0.00010845	0.00069696
Primary Emphysema Pattern: Centrilobular	Fisher exact test	0.00011886	0.00069446
Nodule Attenuation: Solidness More Than 5 mm	Fisher exact test	0.00069605	0.0037278
Nodule Periphery: Presence of emphysema	Fisher exact test	0.00082816	0.0040941
Nodule Associated Findings: Presence of entering airway	Fisher exact test	0.0011145	0.0051163
Nodule Margins: Primary Pattern poorly defined	Fisher exact test	0.0018075	0.0077444
Nodule Margins: Multi-categorical Primary Pattern	Spearman rho	0.0018343	0.0073679

**Table 3:** Top five features for the two analyses; using all non-small cell lung cancers (NSCLC), and focusing only on adenocarcinoma.

Image feature	Percentage selected in N = 100 iterations
**All NSCLC**	
Emphysema: presence	98%
Lung Parenchyma Features: presence of airway abnormality	96%
Nodule Margins: Multi-categorical Primary Pattern	94%
Nodule Attenuation: Multi-class with increasing size of solid component	58%
Nodule Attenuation: Solid	37%
**Adenocarcinoma only**	
Emphysema: presence	93%
Lung Parenchyma Features: presence of airway abnormality	92%
Nodule Margins: Multi-categorical Primary Pattern	92%
Nodule Attenuation: Multi-class with increasing size of solid component	47%
Nodule Attenuation: Solid	44%

**Table 4:** Inter-reader variability of the features in the final model for predicting EGFR mutation status.

Image feature	Cohen’s kappa
Emphysema	0.85
Airway abnormality	0.30
Nodule attenuation	0.16
Nodule margin	0.46
