# Supplementary material for: Predictive radiogenomics modeling of EGFR mutation status in lung cancer
Source: Sci Rep. 2017 Jan 31;7:41674. doi: 10.1038/srep41674 (PMC5282551; doi:10.1038/srep41674)
Supplement: Supplementary Table 1 [file srep41674-s1.pdf]

PREDICTIVE RADIOGENOMICS MODELING OF EGFR MUTATION STATUS IN LUNG CANCER

Olivier Gevaert[1,*], Sebastian Echegaray[2], Amanda Khuong[3], Chuong D. Hoang[3], Joseph B. Shrager[3], Kirstin C. Jensen[4,5], Gerald G. Berry[4], H. Henry Guo[2], Charles Lau[6], Sylvia K. Plevritis[2], Daniel L. Rubin[2], Sandy Napel[2], Ann N. Leung[2]

Supplementary Table 1: Template for capturing all semantic features to study NSCLC.

Supplementary Table 1:

NODULE ANALYSIS

	Anatomic Location	1 = RUL<br>2 = RML<br>3 = RLL<br>4 = LUL<br>5 = Lingula<br>6 = LLL<br>7 = R bronchial tree<br>8 = L bronchial tree<br>9 = Right side<br>10 = Left side<br>99 = Unable to determine
	Axial Location	1 = Central | 2 = Peripheral (edge < 2 cm from visceral pleura)
	Longest diameter (mm)	Integer
	Longest perpendicular diameter (mm)	Integer
	Nodule attenuation	1 = Solid<br>2 = Pure GG (Non-solid)<br>3 = Semi-consolidation (attenuation between solid and ground –glass in non-solid nodules)<br>4 = Part-solid, solid ≤ 5 mm<br>5 = Part-solid, solid > 5 mm
Internal Features	Nodule Reticulation	1 = Absent | 2 = Present (lines inside gg nodule)
	Internal Air alveolograms/bronchograms	1 = Absent | 2 = Present
	Necrosis	1 = Absent | 2 = Present
	Cavitation	1 = Absent | 2 = Present
	Nodule Margins – primary pattern	1 = Smooth (sharply delineated margins – can outline confidently without oscillations or serrations)<br>2 = Irregular (minor oscillations or serrations of margin)<br>3 = Lobulated (focal convexity or protrusions of lesion into lung)<br>4 = Spiculation (several linear radiations of finite length extending into adjacent lung)<br>5 = Poorly defined (lack of clear delineation of margins – cannot outline confidently)
	Nodule Shape	1 = round (roughly spherical)<br>2 = oval (ratio of x/y diameters >1.5)<br>3 = complex (neither 1 nor 2)<br>4 = polygonal (straight or concave borders)
	Nodule Calcification	1 = No calcification<br>2 = Central calcification<br>3 = Peripheral
Associated Findings	Attachment to Pleura	1 = Absent | 2 = Present
	Attachment to Vessel	1 = Absent | 2 = Present
	Attachment to Bronchus	1 = Absent | 2 = Present
	Pleural Retraction	1 = Absent | 2 = Present
	Entering Airway	1 = Absent | 2 = Present
	Thickened adjacent bronchovascular bundle	1 = Absent | 2 = Present
	Vascular convergence	1 = Absent | 2 = Present
	Septal thickening	1 = Absent | 2 = Present
	Nodule Periphery	1 = Emphysema<br>2 = Fibrosis (diffuse)<br>3 = Normal<br>4 = Scarring (focal)
	Satellite nodules in Primary Lesion Lobe (≥ 4mm, noncalcified)	1 = Absent | 2 = Solid | 3 = Non-solid | 4 = Semi-consolidation | 5 = Part-solid
	Nodules in NON-lesion lobe SAME Lung (≥ 4mm, noncalcified)	1 = Absent | 2 = Solid | 3 = Non-solid | 4 = Semi-consolidation | 5 = Part-solid

	Nodules in CONTRALATERAL Lung (≥ 4mm, noncalcified)	1 = Absent | 2 = Solid | 3 = Non-solid | 4 = Semi-consolidation | 5 = Part-solid
	Centrilobular nodules – diffuse (RB type nodules)	1 = Absent | 2 = Present

LUNG PARENCHYMA ANALYSIS

	Emphysema	1 = Absent | 2 = Present
	Primary emphysema Pattern	1 = Centrilobular<br>2 = Pan-acinar<br>3 = Paraseptal<br>4 = Paracicatricial<br>99 = NA
	Primary Distribution	1 = Upper predominant<br>2 = Middle Predominant<br>3 = Lower Predominant<br>4 = Diffuse, no predominance<br>5 = Patchy, no predominance<br>99 = NA or Unable to determine
	Primary Emphysema Laterality	1 = Right | 2 = Left 3=Both
	Secondary Emphysema Pattern	1 = Centrilobular<br>2 = Pan-acinar<br>3 = Paraseptal<br>4 = Paracicatricial<br>99 = NA
	Secondary Emphysema Distribution	1 = Upper predominant<br>2 = Middle Predominant<br>3 = Lower Predominant<br>4 = Diffuse, no predominance<br>5 = Patchy, no predominance<br>99 = NA or Unable to determine
	Secondary emphysema laterality	1 = Right | 2 = Left 3= Both
	Overall Emphysema Severity	0 = None<br>1 = Low (1-25%)<br>2 = Moderate (26-50%)<br>3 = Moderately High (51-75%)<br>4 = High (> 75%)
Lung Features	Airway Abnormalities	1 = Absent | 2 = Present
	Bronchial wall thickening	1 = Absent | 2 = Present
	Airway ectasia (mild luminal enlargement)	1 = Absent | 2 = Present
	Bronchiectasis (moderate enlargement)	1 = Absent | 2 = Present
	Luminal narrowing	1 = Absent | 2 = Present
	Bronchiolar prominence	1 = Absent | 2 = Present
	Tree-in-Bud (airway secretions)	1 = Absent | 2 = Present
	Mosaic oligemia	1 = Absent | 2 = Present
	Fibrosis	0 = Absent | 1 = Present
	Anatomic Fibrosis Distribution	1 = Apical<br>2 = Upper predominant<br>3 = Middle Predominant<br>4 = Lower Predominant<br>5 = Diffuse, no predominancne<br>6 = Patchy, no predominance<br>99 = Unable to determine
	Axial Fibrosis Distribution	1 = Subpleural<br>2 = Bronchovascular<br>3 = Both 1 & 2<br>4 = Random
	Fibrosis Type	1 = UIP<br>2 = NSIP<br>3 = HP<br>4 = Sarcoidosis

		5 = Smoking-related<br>6 = Post-infectious (include OGD)<br>7 = Other (specify)<br>8 = Indeterminate

Adds: longest solid diameter (mm)

% Ground glass: 1= 1-25%; 2=26-50%; 3= 51-75%; 4=76-100%

http://epad-prod3.stanford.edu:8080/epad/`
